# Effectiveness and safety of mineralocorticoid receptor antagonists in heart failure patients with and without diabetes: a systematic review and meta-analysis

**DOI:** 10.1186/s43044-024-00580-5

**Published:** 2024-11-14

**Authors:** Arga Setyo Adji, Jordan Steven Widjaja, Bryan Gervais de Liyis

**Affiliations:** 1 Faculty of Medicine, Hang Tuah University, Ahmad Yani Street no.1, Wonokromo, Surabaya, East Java 60244 Indonesia; 2 Faculty of Medicine, Udayana University, Denpasar, Bali Indonesia

**Keywords:** Heart failure, Diabetes mellitus, Mineralocorticoid receptor antagonists, Steroid

## Abstract

**Background:**

Mineralocorticoid receptor antagonists (MRAs) have been shown to improve outcomes in various populations of heart failure (HF) patients. However, the impact of concomitant diseases, such as diabetes mellitus (DM), on these outcomes remains unclear. This meta-analysis aimed to evaluate the efficacy and safety of MRAs in heart failure patients with and without diabetes mellitus.

**Methods:**

A systematic search was conducted on PubMed, Scopus, and Google Scholar databases up to April 30, 2024. Data analysis was performed using a random-effects model to account for variability across studies, and statistical analysis was carried out using Review Manager 5.4. Efficacy and safety parameters were evaluated in accordance with the Preferred Reporting Items for Systematic Review and Meta-Analysis guidelines.

**Results:**

The meta-analysis included a total of 21,832 subjects from ten studies. The pooled results demonstrated that MRAs, compared to placebo, significantly reduced all-cause mortality in HF patients with and without DM (RR: 0.85; 95%CI 0.75–0.96; *p* = 0.009). A similar effect was observed in HF patients without DM (RR: 0.83; 95%CI 0.71–0.97; *p* = 0.02), while no significant effect was detected in the DM subgroup (RR: 0.87; 95%CI 0.69–1.11; *p* = 0.27). Both treatments had comparable effects on cardiovascular mortality in HF patients with and without DM (RR: 0.88; 95%CI 0.82–0.94; *p* = 0.0002), in HF patients with DM (RR: 0.90; 95%CI 0.81–1.01; *p* = 0.08), and in the non-DM subgroup (RR: 0.86; 95%CI 0.79–0.94; *p* = 0.0009). MRAs significantly reduced the risk of cardiovascular mortality in HF patients with and without DM (RR: 0.82; 95%CI 0.72–0.94; *p* = 0.005) and in HF patients with DM (RR: 0.79; 95%CI 0.63–0.98; *p* = 0.03), but no significant effect was observed in the non-DM subgroup (RR: 0.85; 95%CI 0.69–1.05; *p* = 0.13). Furthermore, compared to placebo, MRAs were associated with an increased risk of hyperkalemia (> 5.5 mEq/L) in HF patients with and without DM (RR: 1.63; 95%CI 1.18–2.24; *p* = 0.003), particularly in HF patients with DM (RR: 1.44; 95%CI 0.97–2.13; *p* = 0.07) and in the non-DM subgroup (RR: 1.87; 95%CI 1.34–2.61; *p* = 0.0002).

**Conclusion:**

MRAs are effective in reducing all-cause mortality, cardiovascular death, and cardiovascular mortality in heart failure patients. However, the use of MRAs is associated with an increased risk of hyperkalemia, necessitating careful monitoring, particularly in patients with diabetes mellitus.

## Background

According to the European Society of Cardiology (ESC 2016), the most noticeable symptoms of heart failure are shortness of breath, swollen ankles, and excessive fatigue. In addition to a reduction in cardiac output and/or higher intracardiac pressure at rest and under stress, other symptoms, such as peripheral edema, pulmonary crackles, and raised jugular venous pressure, may suggest a structural or functional cardiac abnormality [1]. It is possible to diagnose heart failure when symptoms start to show. Heart failure cannot be diagnosed without first identifying an underlying cardiac condition. The systolic and/or diastolic ventricles often become dysfunctional due to a cardiac abnormality, such as a myocardial infarction. A variety of cardiac rhythm and conduction disorders, as well as those affecting the pericardium and endocardium, as well as the valves (stenosis and regurgitation), may lead to heart failure [2].

Studies conducted in the USA have shown that ischemic heart disease, hypertension, diabetes mellitus, advanced age (> 65 years), and obesity are the primary risk factors for the development of HF [3]. Similar risk variables were also found in studies from European nations; however, smoking was included as a primary risk factor for heart failure (HF) [4, 5]. Changes in glycemic status are frequently linked to other cardiovascular risk factors, including obesity, dyslipidemia, and hypertension. These variables are early risk factors for the onset of HF and have been underlined in the revised definition of HF [6]. T2DM alone may hasten the development of extracellular matrix collagen deposition, coronary and systemic atherosclerosis, vascular alterations, and autonomic dysfunction [7, 8].

There are other ways that type 2 diabetes can impact the structure and function of the heart, but the most significant way is related to insulin resistance in muscle, liver, and pancreatic cells. In these systems, the absence of an insulin response results in decreased levels of incretin from the gastrointestinal tract, increased renal glucose absorption, faster lipolysis, systemic glucotoxicity, and fatty acid lipotoxicity. Notably, cardiac damage can result from a variety of changes, including endothelial (increased RAA activity, vascular growth factors, and decreased NO synthase), metabolic (lipogenesis and gluconeogenesis), renal (increased Na and glucose resorption), myocardial (sarcomeric stiffness and fibrosis overexpression), and inflammatory disorders (increased expression of interleukins facilitating thrombogenesis). The various HF patterns and heart structural adaptations may be explained by the predominance of each pathological cause [8]. The prognosis for hospitalized diabetes mellitus DM patients with HF is significantly worse, with higher rates of post-discharge HF hospitalization and cardiovascular (CV) death [9]. According to a recent subgroup analysis of the data, hospitalized HF patients with DM had a greater likelihood of experiencing adverse effects during conventional treatment than did patients without DM [10]. As a result, managing concurrent HF and DM remains difficult [11].

Patients with CVD can benefit greatly from the use of mineralocorticoid receptor antagonists (MRAs) as a treatment [12]. MRA therapy has been shown to reduce morbidity and mortality in HF patients, and as a result, MRAs are now a regular component of HF treatment [13, 14]. Treatment with MRAs is linked to better outcomes in patients with DM, similar to what has been observed in HF patients without DM [15]. It is imperative to acknowledge the potential side effects of hyperkalemia, gynecomastia, irregular menstruation, and acute renal injury [16]. However, how MRAs affect glycemic regulation is unclear. While spironolactone has been linked to significant increases in HbA1c levels and worsening glycemic control in some studies [17, 18], a study found that spironolactone may benefit patients with nonalcoholic fatty liver disease in terms of serum insulin and homeostatic model assessment for insulin resistance (HOMA-IR) [19]. The idea that MRAs—spironolactone or eplerenone—did not significantly alter glucose levels is supported by a few studies [20–22]. Moreover, spironolactone increased HbA1c in individuals with DM and HF, while eplerenone did not, according to the findings of a small direct comparison experiment [23]. It is necessary to gain further insight into the safety and effectiveness of MRAs in patients with DM and HF. No meta-analysis has been performed to date on the association between MRA treatment and patient outcomes. Consequently, it is logical to conduct a systematic review to evaluate the safety and effectiveness of MRA treatment in patients who both have DM and HF [11].

## Methods

Consistent with the PRISMA guidelines, this systematic review gathered and analyzed relevant studies [24].

### Eligibility criteria

In this systematic review, studies meeting specific inclusion and exclusion criteria were considered for analysis. The included studies were required to compare the efficacy and safety of mineralocorticoid receptor antagonists against placebo in heart failure patients with and without diabetes mellitus. Additionally, eligible studies were expected to report outcome measures such as (1) efficacy (all-cause mortality, CV mortality, and all mortality/CV hospitalization), (2) safety (hyperkalemia > 5.5 mEq/L), and (3) randomized controlled trial (RCT) study designs; additionally, (4) written in English was used. Conversely, studies not comparing MRA against placebo or those lacking relevant outcome measures were excluded from consideration. Furthermore, nonhuman studies were also excluded from the analysis to ensure the relevance and applicability of the findings to the target population of HF patients.

### Search strategy and selection of studies

From April 2024 onward, relevant subjects were identified by searching many other databases, including PubMed, ScienceDirect, and Google Scholar. The formula search used Boolean “AND” or “OR” with “MRA,” “T2DM,” “heart failure,” “diabetes mellitus,” “diabetes,” “canrenone,” “spironolactone,” “hyperkalemia,” “aldosterone,” “placebo,” and “eplerenone.” Furthermore, to find more relevant and comparable research, we examined the references included in the identified papers.

### Data extraction

Upon selection of relevant studies, the extraction of pertinent data was meticulously carried out by designated investigators (A.S.A. and J.S.W.) utilizing a predefined data extraction form. The extracted data encompassed various aspects, including study characteristics such as author year, study design, study periods, location, population (MRAs and placebo), mean age ± SD, NYHA function class, EF (%), and eGFR (mL/min per 1.73 m^2^). To maintain the integrity and precision of the data extraction process, a thorough cross-checking procedure was implemented. Another investigator independently reviewed the extracted data to verify its accuracy and completeness, thereby mitigating the risk of errors or omissions. This stringent validation process ensured the reliability and robustness of the extracted data for subsequent analysis.

### Quality assessment

We conducted an exhaustive evaluation of potential bias using the Cochrane Risk of Bias (RoB) Tool, which includes a seven-step method for assessing bias as recommended by the Cochrane Collaboration [25]. Critical components such as participant blinding, allocation concealment, randomization procedures, insufficient outcome data, selective reporting, and other types of bias were investigated in depth to ascertain the possibility of bias in the studies. J.S.W. and A.S.A. were the quality assessors. All parties involved in this evaluation are committed to working together to resolve any disputes that may emerge.

### Outcome measure

The analysis considered several outcome measures, encompassing efficacy and safety. Efficacy was evaluated in terms of all-cause mortality, death from CV, and CV mortality from hospitalization for HF, while safety endpoints included hyperkalemia > 5.5 mEq/L.

### Data synthesis and statistical analysis

Pooled risk ratios (RRs) and 95% CIs for each outcome measure were calculated in this research via a meta-analysis. Using the I^2^ statistic, we reviewed the included studies for heterogeneity. It is possible to perform subgroup analysis by taking into account whether heart failure patients have diabetes mellitus. Furthermore, to assess how resilient the findings are, sensitivity analysis will be carried out. A significance level of *p* < 0.05 was established. Review Manager 5.4 was used to perform the statistical analyses [26].

## Results

### Study selection process and quality assessment

**Fig. 1:**
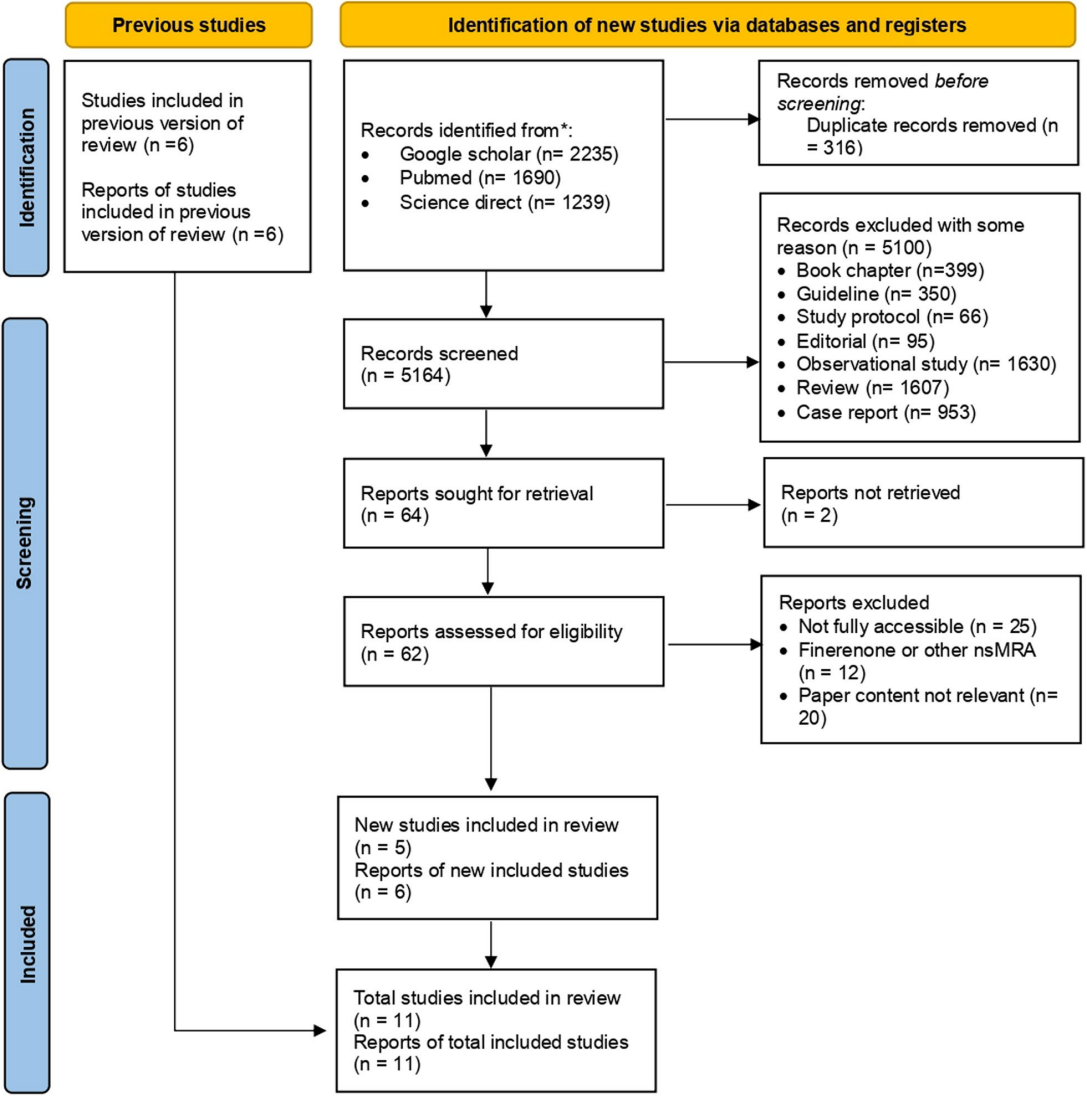
PRISMA flow diagram of the study selection process

The search yielded 5164 records from Google Scholar (*n* = 2235), PubMed (*n* = 1690), and ScienceDirect (*n* = 1239), with 316 duplicates removed. After screening titles and abstracts, 5100 records were excluded, including book chapters (*n* = 399), guidelines (*n* = 350), study protocols (*n* = 66), editorials (*n* = 95), observational studies (*n* = 1630), reviews (*n* = 1607), and case reports (*n* = 953). Among the 64 reports sought for retrieval, two could not be retrieved. Full-text screening of the remaining 62 reports resulted in the exclusion of 25 due to inaccessibility, 12 involving eplerenone or other non-MRA drugs, and 20 for irrelevance. Ultimately, five new studies were included in the review, bringing the total number of included studies to 10. A PRISMA flowchart summarizing the study selection process is provided in Fig. 1.

### Study characteristics

The association between HF with or without DM and our outcomes of interest was examined in this meta-analysis, which was drawn from ten studies including 21,832 individuals (Table 1). There were a total of five studies from multiple sites (*n* = 5); the next most common regions were Europe (*n* = 3), the US (*n* = 1), and Asia (*n* = 1). The length of the follow-up ranged from 6 to 48 months.

**Table 1:** Data characteristics

No.	Author, year	Study design	Study periods	Location (country)	Population		Mean age ± SD	NYHA function class	EF (%)	eGFR (mL/min per 1.73 m^2^)
					MRA	Placebo				
1	Pitt et al. [27]	RCT	1995–1998	Multicenter	822	841	65 ± 12 both of group	NYHA class III and IV	≤ 35%	N.A
2	Pitt et al. [28]	RCT	1999–2001	Multicenter	3319	3313	64 ± 11 for eplerenone and 64 ± 12 for placebo	N.A	≤ 40%	N.A
3	O’Keefe et al. [29]	RCT	2005	USA	749	734	60 ± 10 both of group	N.A	≤ 40%	N.A
4	Boccanelli et al. [30]	RCT	2007–2008	Italy	231	236	62.3 ± 9.5 for canrenone and 62.7 ± 9.5 for placebo	NYHA class II	≤ 45%	N.A
5	Zannad et al. [31]	RCT	2006–2010	Multicenter	1364	1373	68.7 ± 7.7 for eplerenone and 68.6 ± 7.6 for placebo	NYHA class II	30–35%	≥ 30 mL/min per 1.73 m^2^
6	Eschalier et al. [32]	RCT	2006–2012	France	459	400	68.1 ± 7.4 for eplerenone and 68.6 ± 7.6 for placebo	NYHA class II	30–35%	≥ 30 mL/min per 1.73 m^2^
7	Pitt et al. [33]	RCT	2006–2012	Multicenter	1722	1723	68.7 both of group	NYHA class I–IV	≥ 45	≥ 30 mL/min per 1.73 m^2^
8	Vaduganathan et al. [34]	RCT	2003–2006	Multicenter	444 for diabetic and 801 for non-diabetic	306 for diabetic and 447 for non-diabetic	65.1 ± 10.1 for MRA, 67.9 ± 10.7 for placebo in diabetic and 63.6 ± 12.8 for MRA, 67.6 ± 12.6 for placebo in non-diabetic	NYHA class IV	≤ 40%	< 60 mL/min per 1.73 m^2^
9	Vizzardi et al. [35]	RCT	2001–2004	Italy	65	65	61 ± 14.7 for spironolactone and 65 ± 17.4 for placebo	NYHA class I and II	< 40%	≥ 30 mL/min per 1.73 m^2^
10	Tsutsui et al. [36]	RCT	2010–2015	Japan	111	110	69.0 ± 8.7 for eplerenone and 68.4 ± 7.7 for placebo	NYHA class II–IV	≤ 35%	≥ 30 mL/min per 1.73 m^2^
11	Ferreira et al. [37]	RCT	2017–2020	Multicenter	1355	512	65.7 ± 11.1 for MRA user and 69.6 ± 10.5 for no MRA user	NYHA class II–IV	≤ 30%	≥ 30 mL/min per 1.73 m^2^

### Risk of bias

**Fig. 2:**
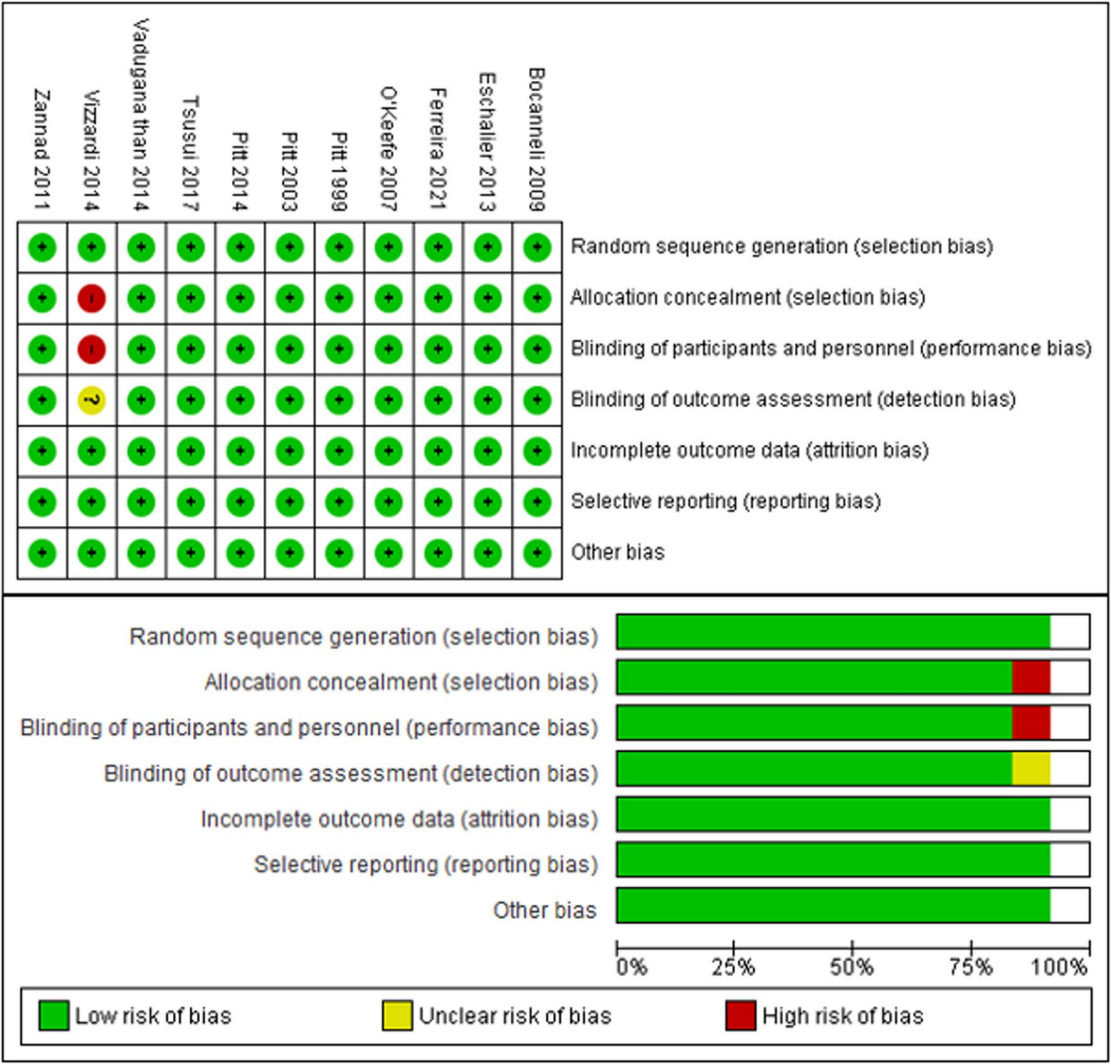
Risk of bias summary and graph

**Fig. 3:**
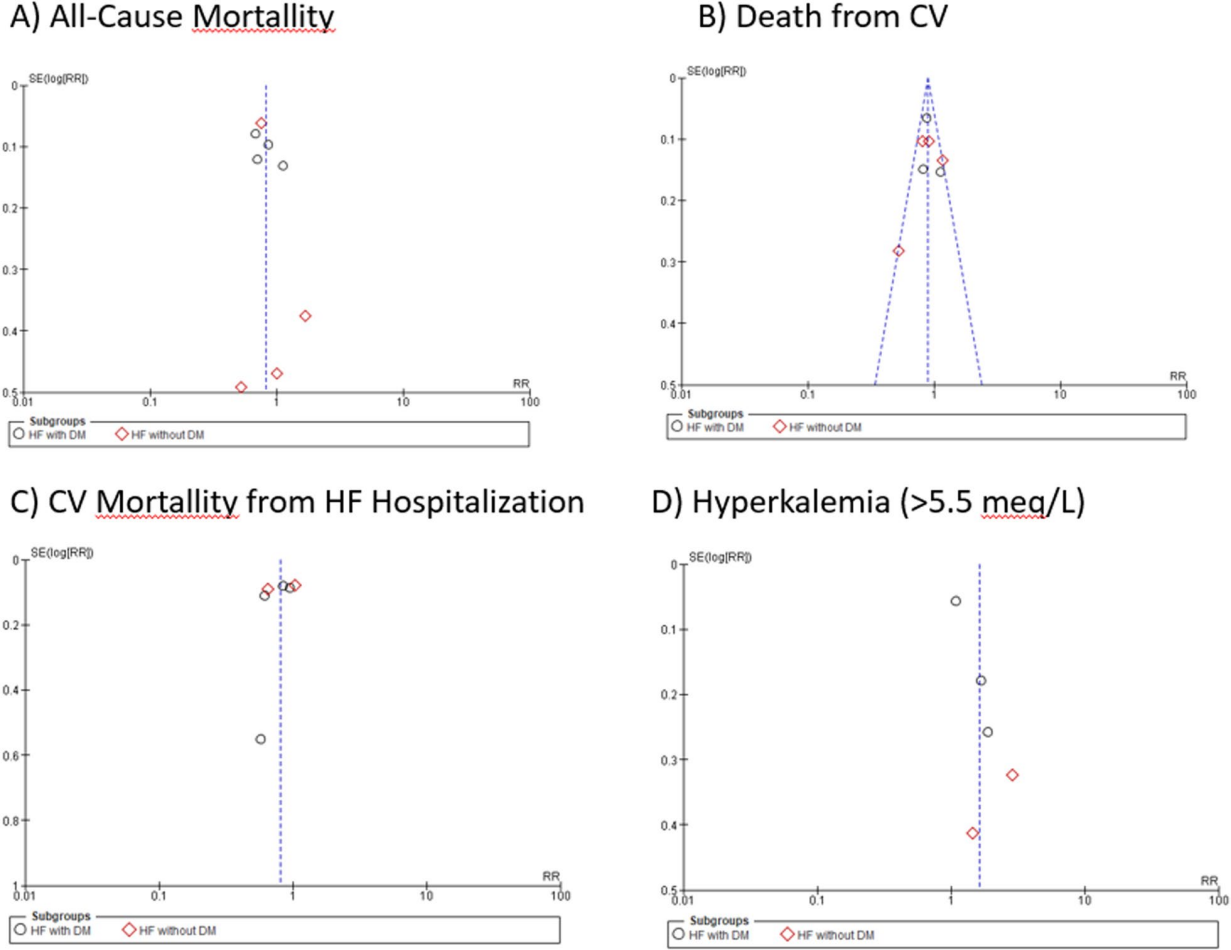
Funnel plot of publication bias for **A** all-cause mortality, **B** death from CV, **C** CV mortality from hospitalization for HF, and **D** hyperkalemia (> 5,5 mEq/L)

The ten included studies were classified as having a different risk of bias according to the method used (Table 2). All studies were assessed by RoB, and all of the studies were classified as having a low risk of bias, which indicates that the studies included are of high quality. Based on the results of the Cochrane risk of bias review, all of the included studies were considered to have low bias quality (Fig. 2). There was a substantial likelihood of performance bias in all of the investigations [28, 30–32, 34, 35, 37–40]. Nevertheless, it should be mentioned that every single study was classified as having an uncertain bias, especially in the area of detection bias, which is caused by variables that impact the evaluation of the results that cannot be explained. We need to find bias in each result. Figure 3 shows that there was a minimal probability of bias (*I*^2^ = 41%) since the funnel plots for mortality from CV outcomes were symmetrical. The findings of the included studies were found to vary, with funnel plots for all-cause mortality, CV mortality following hospitalization for HF, and hyperkalemia displaying asymmetry (*I*^2^ = 52%, *I*^2^ = 77%, and *I*^2^ = 79%).

**Table 2:** Study outcome

No.	Author, year	Drug comparator	Treatment duration	Main result	Key outcomes	Study quality
1	Pitt et al. [27]	I: Spironolactone C:Placebo	24 months	When given to individuals suffering from severe heart failure, spironolactone produced remarkable improvements in their health. The specific number of patients whose conditions improved, remained unchanged, or deteriorated in the spironolactone group was 41%. A Wilcoxon test result of less than 0.001 indicates that this group difference is statistically significant. In addition, the study was stopped before it was finished because the expected critical z value for the effect of spironolactone on the risk of death from any cause was greater than what was discovered (2.02, which is comparable to a P value of 0.043)	Spironolactone improved outcomes in 41% of severe heart failure patients, and the study was stopped early due to a reduced risk of death	Good
2	Pitt et al. [28]	I: Eplerenone C:Placebo	24 months	Eplerenone, when added to optimal medical therapy, decreases morbidity and mortality in patients suffering from acute myocardial infarction complicated by left ventricular dysfunction and heart failure. It specifically lowered the risk of death from cardiovascular causes or hospitalization for cardiovascular events (relative risk, 0.87; *P* = 0.002) and reduced the incidence of sudden cardiac death (relative risk, 0.79; *P* = 0.03)	Eplerenone’s reduction in cardiovascular mortality was primarily driven by a 21% decrease in sudden cardiac death and a 15% reduction in the risk of hospitalization due to heart failure	Good
3	O’Keefe et al. [29]	I: Eplerenone C: Placebo	16 months	When comparing the eplerenone group to the placebo group in diabetic people, the relative risk was 0.83 (*p* = 0.031), and the incidence of cardiovascular death or hospitalization was 35.8% versus 40.9%. Other causes of death, such as cardiovascular disease, were also not significantly different among the individuals. Remarkably, compared to the non-diabetic sample, the diabetic subgroup had a 5.1% absolute risk reduction for cardiovascular hospitalization and mortality, which was superior	Eplerenone reduced cardiovascular death or hospitalization by 5.1% in diabetic patients, showing greater benefit than in non-diabetic patients	Good
4	Boccanelli et al. [30]	I: Canrenone C:Placebo	12 months	Compared to placebo, canrenone substantially improved outcomes in stabilized heart failure patients in NYHA class II by lowering left ventricular mass (*P* = 0.02). Canrenone also altered cardiac geometry in a favorable way, as reflected by a greater reduction in left atrium size. Canrenone reduced hospitalizations for heart disease and worsening heart failure, and significantly reduced the feasibility of cardiac death or hospital admissions, with 7.9% of patients versus 15.1% (*P* = 0.02)	Canrenone reduced cardiac mass, improved heart geometry, and halved cardiac death or hospitalization rates	Good
5	Zannad et al. [31]	I: Eplerenone C:Placebo	21 months	With a hazard ratio of 0.76 (95% CI, 0.62–0.93), eplerenone considerably decreased the likelihood of cardiovascular-related hospitalizations or deaths. The enormous advantage (*P* < 0.001) led to the early termination of the experiment. The major and secondary outcomes’ adjusted P values were often less than 0.001, suggesting that the eplerenone and placebo groups differed significantly. From 33 (2.4% of the total) to 569 (41.4%), people would require treatment each year to avoid a single major result	Eplerenone reduced cardiovascular hospitalizations or deaths, prompting early trial termination	Good
6	Eschalier et al. [32]	I: Eplerenone C:Placebo	6 months	There was a specified incidence of discontinuation owing to adverse events throughout the whole trial population and high-risk subgroups for eplerenone compared to placebo at month 5, and there was also a standardized mean dosage. According to Kaplan–Meier curves, the hazard ratios for eplerenone compared to placebo for the main composite endpoint—which includes hospitalization for heart failure (HF) or death from cardiovascular causes—were revealed. The fact that it produces these results proves that it is effective. The evaluation process also included a thorough examination of subgroup analyses and baseline characteristics	Eplerenone caused more discontinuations due to adverse events and effectively reduced HF hospitalizations or cardiovascular deaths	Good
7	Pitt et al. [33]	I: Spironolactone C:Placebo	36 months	The primary outcome, which is a composite of death from cardiovascular causes, aborted cardiac arrest, or hospitalization for heart failure, did not show a statistically significant reduction in incidence rates with 5.9 vs 6.6 occurrences per 100 person-years, *P* = 0.14. Without adjusting for confounding factors, the hazard ratio came to 0.89 (95% CI, 0.77–1.04). The most common component, hospitalization for heart failure, was less common in the spironolactone group	Spironolactone did not significantly reduce cardiovascular events but reduced heart failure hospitalizations	Good
8	Vaduganathan et al. [34]	I: Canrenoic acid, canrenone, potassium canreonate, eplerenone, soludactone, and spironolactone C:Placebo	9.9 months	Although 62.3% of patients had MRA done at discharge, the percentage was 64.2% for patients without DM and 59.2% for those with DM. There is statistical research that backs up this notion. The primary results were mortality from any cause, mortality from cardiovascular disease, and hospitalization due to heart failure. The chi-square, Fisher’s exact, and Kruskal–Wallis tests were among the many statistical methods used. A log-rank test and Cox proportional hazard models were used to assess the data on time-to-event	MRA use in diabetes mellitus patients showed no significant impact on mortality or cardiovascular outcomes compared to non-diabetic patients, with overall MRA initiation remaining low	Good
9	Vizzardi et al. [35]	I: Spironolactone C:Placebo	44 ± 16 months	There was no correlation between the use of spironolactone, LVEF, or blood creatinine levels and the risk of cardiovascular death or hospitalization. Treatment with spironolactone and levels of creatinine were shown to be statistically significant in the context of hospitalizations for cardiovascular conditions. The average duration of follow-up for the 130 patients who were given spironolactone or a placebo at random was 44 ± 16 months. Thirty individuals were admitted to the hospital because of heart problems, eighteen for heart failure, seven for fatalities caused by heart problems, and nine for reasons unrelated to heart problems	Spironolactone use, LVEF, and creatinine levels showed no correlation with cardiovascular death or hospitalization risk, though spironolactone significantly impacted hospitalization rates	Good
10	Tsutsui et al. [36]	I: Eplerenone C:Placebo	48 months	Eplerenone did not significantly lower cardiovascular-related hospitalizations or deaths in the Japanese patients compared to the placebo group in the J-EMPHASIS-HF study. In all, 221 patients participated in the study, with a median follow-up duration of 862 days. Eplerenone had comparable side effects, such as hyperkalemia, to the placebo group, and it was well-tolerated. Additional study is needed to validate its efficacy; however, it shown positive benefits on LVEF levels, plasma BNP, and hospitalization for any reason	Eplerenone did not significantly reduce cardiovascular-related hospitalizations or deaths compared to placebo but improved LVEF, BNP levels, and overall hospitalization rates	Good
11	Ferreira et al. [37]	I: MRA C: Placebo	27 months	The main results include a treatment effect with hazard ratios and odds ratios indicating the effectiveness of the treatment across various metrics, such as a treatment effect of 0.76 (0.59–0.97) and 0.69 (0.48–0.97) for certain outcomes. Interaction p values ranged from 0.10 to 0.93, suggesting varying levels of statistical significance across different interactions. After controlling for a number of factors, logistic regression was used to compare the percentage of responders across many therapy groups	Empagliflozin’s effectiveness in heart failure was unaffected by MRA use, reducing both MRA initiation and discontinuation, and leading to fewer severe hyperkalemia cases	Good

### Efficacy and safety of mineralocorticoid receptor antagonists vs placebo

#### All-cause mortality

**Fig. 4:**
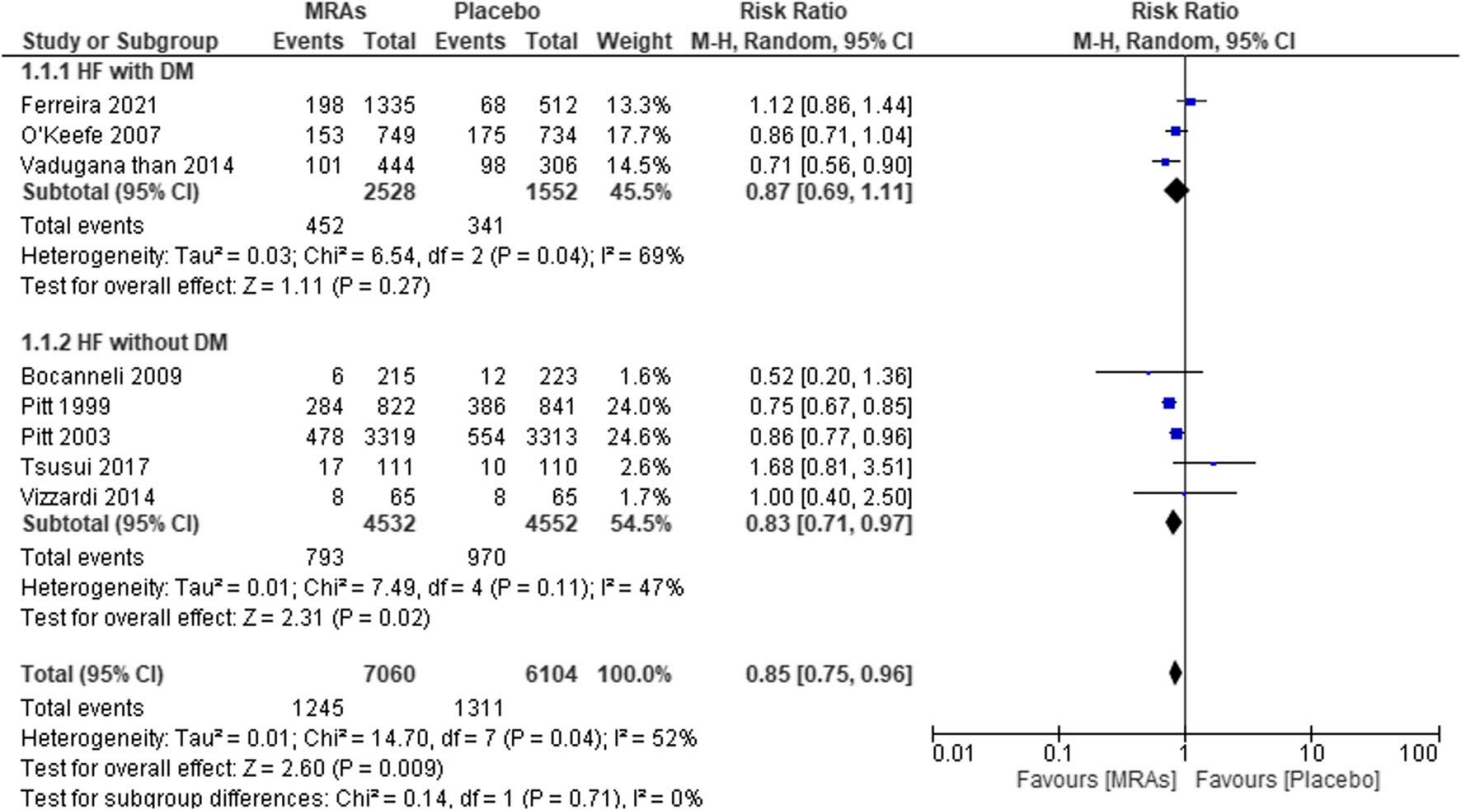
Forest plot of all-cause mortality in patients treated with MRAs vs placebo

In Fig. 4, a total of eight studies represented the total number of participants in each treatment group across all the included studies. There were 4532 participants in the studies treated with MRAs and 4552 participants treated with placebo. Our pooled analysis confirmed a significant difference between the MRA and placebo groups, with a pooled RR of 0.85 (95% CI 0.75–0.96, *P* = 0.009; *I*^2^ = 52%). The MRA group exhibited a significantly lower rate of all-cause mortality in HF with DM participants (RR 0.87; 95% CI [0.69–1.11]; *P* = 0.27; I^2^ = 69%) and an insignificantly lower RR in HF without DM participants (RR 0.83; 95% CI [0.71–0.97]; *P* = 0.02; *I*^2^ = 47%) (Table 3).

**Table 3:** Summary of results

End point	DM M:P RR (95% CI)	N-DM M:P RR (95% CI)	*p*-value	EF categories		
				HFmrEF M:P RR (95% CI)	HFrEF M:P RR (95% CI)	*p*-value
*Efficacy*						
All-cause mortallity	0.87 (0.69–1.11)	0.83 (0.71–0.97)	0.009^*^	0.87 (0.73–1.04)	0.84 (0.70–1.02)	0.009^*^
Death from CV	0.90 (0.81–1.01)	0.86 (0.79–0.94)	0.0002 ^*^	0.95 (0.83–1.08)	0.73 (0.43–1.25)	0.19
CV mortallity from HF hospitalization	0.79 (0.63–0.98)	0.85 (0.69–1.05)	0.005^*^	0.78 (0.62–0.97)	0.58 (0.20–1.69)	0.02^*^
*Safety*						
Hyperkalemia	1.44 (0.97–2.13)	1.87 (1.34–2.61)	0.003^*^	1.43 (1.01–2.01)	2.05 (1.26–3.36)	0.003^*^

CI, Confidence interval; HFmrEF, Heart failure with mildly reduced ejection fraction; HFrEF, Heart failure with reduced ejection fraction; M: MRAs, Steroidal mineralocorticoid receptor antagonist; P, Placebo; DM, Diabetes mellitus: N-DM, Non-diabetes mellitus; and RR, Risk ratio

*indicating statistical significance

#### Death from CV

**Fig. 5:**
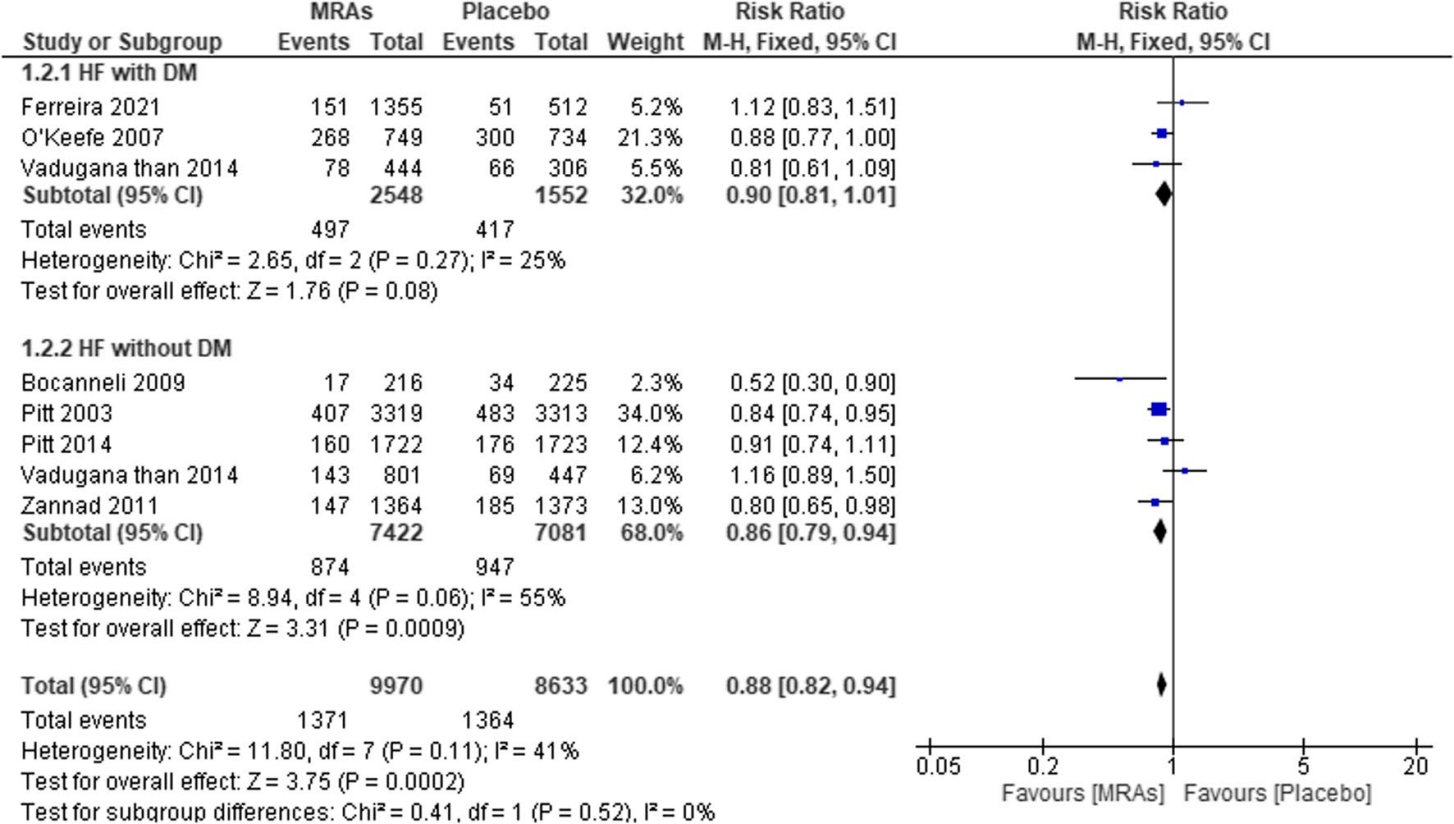
Forest plot of mortality from CVD for patients treated with MRAs vs placebo

Six studies examined the risk ratio of cardiovascular death in heart failure patients with and without diabetes mellitus to that of patients receiving placebo or MRAs; the findings are shown in Fig. 5. After combining all of the data, we found that MRA treatment significantly lowered the risk of death from CV causes in HF patients without “DM” (RR 0.90, 95% CI 0.81–1.01, *P* = 0.08, *I*^2^ = 25%) but only marginally in HF patients with “DM” (RR 0.88, 95% CI 0.82–0.94, *P* = 0.0002, *I*^2^ = 41%). The risk of death from cardiovascular causes was significantly lower in the group that received MRAs than in the placebo group (RR = 0.86, 95% CI 0.79–0.94; *P* = 0.0009, *I*^2^ = 55%).

#### CV mortality from HF hospitalization

**Fig. 6:**
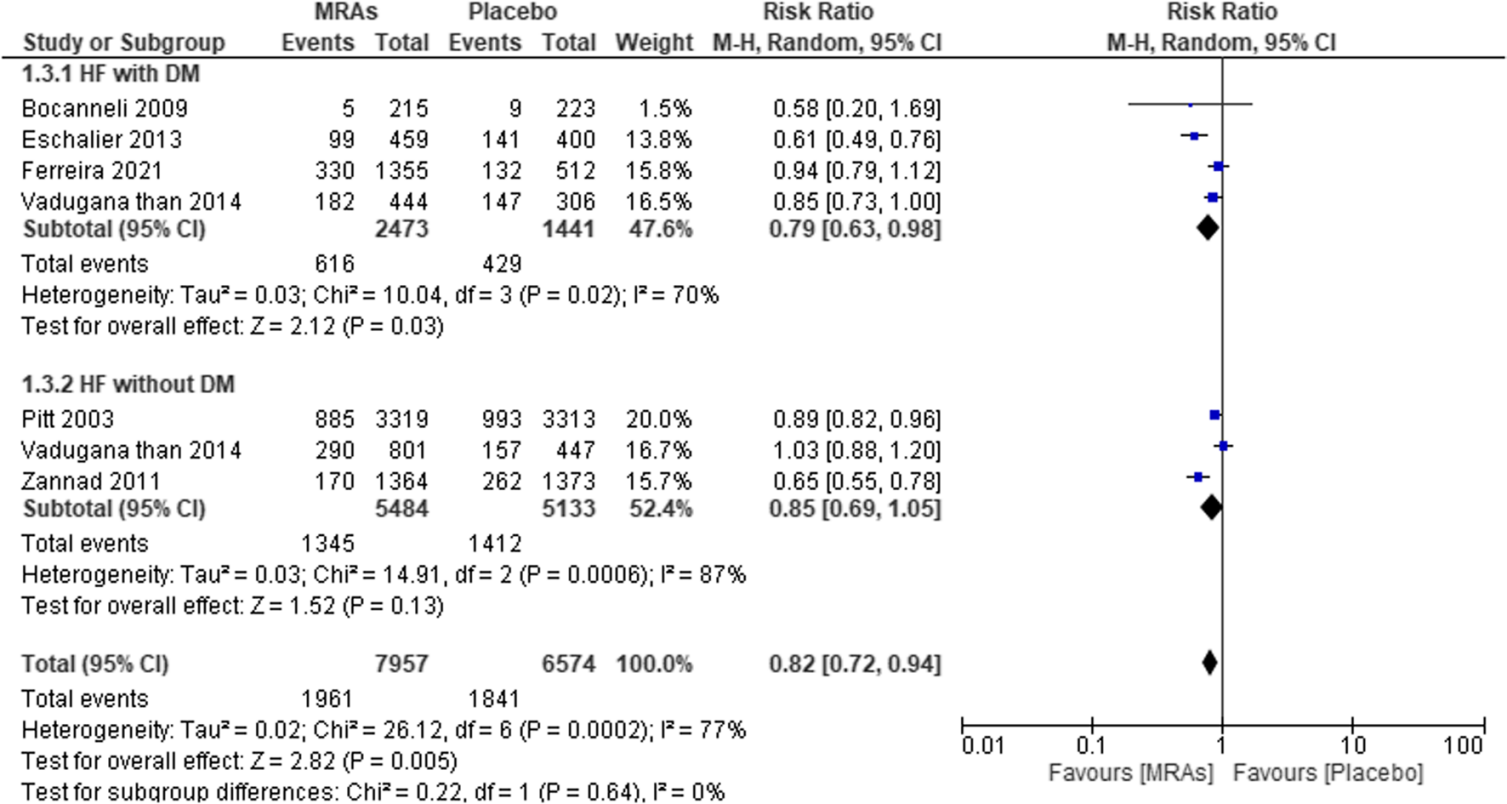
Forest plot of mortality due to HF hospitalization in patients treated with MRAs vs placebo

Relative to the placebo, MRAs were linked to a reduced risk of death from HF hospitalization in both DM and non-DM patients, as shown in Fig. 6, which summarizes the findings of five studies. With a pooled RR of 0.82 (95% CI 0.72–0.94, *P* = 0.005, *I*^2^ = 77%), the MRA group was shown to have a significantly lower risk ratio than the placebo group. For heart failure patients with diabetes mellitus (RR = 0.79, 95% CI = 0.63–0.98, *P* = 0.03, *I*^2^ = 70%), compared with placebo, MRAs significantly decreased the risk of diabetes mellitus. However, for heart failure patients without diabetes mellitus (RR = 0.85, 95% CI = 0.69–1.05; *P* = 0.13, *I*^2^ = 87%), the reduction in risk was not statistically significant.

### Safety of mineralocorticoid receptor antagonists vs placebo

#### Hyperkalemia (> 5.5 mEq/l)

**Fig. 7:**
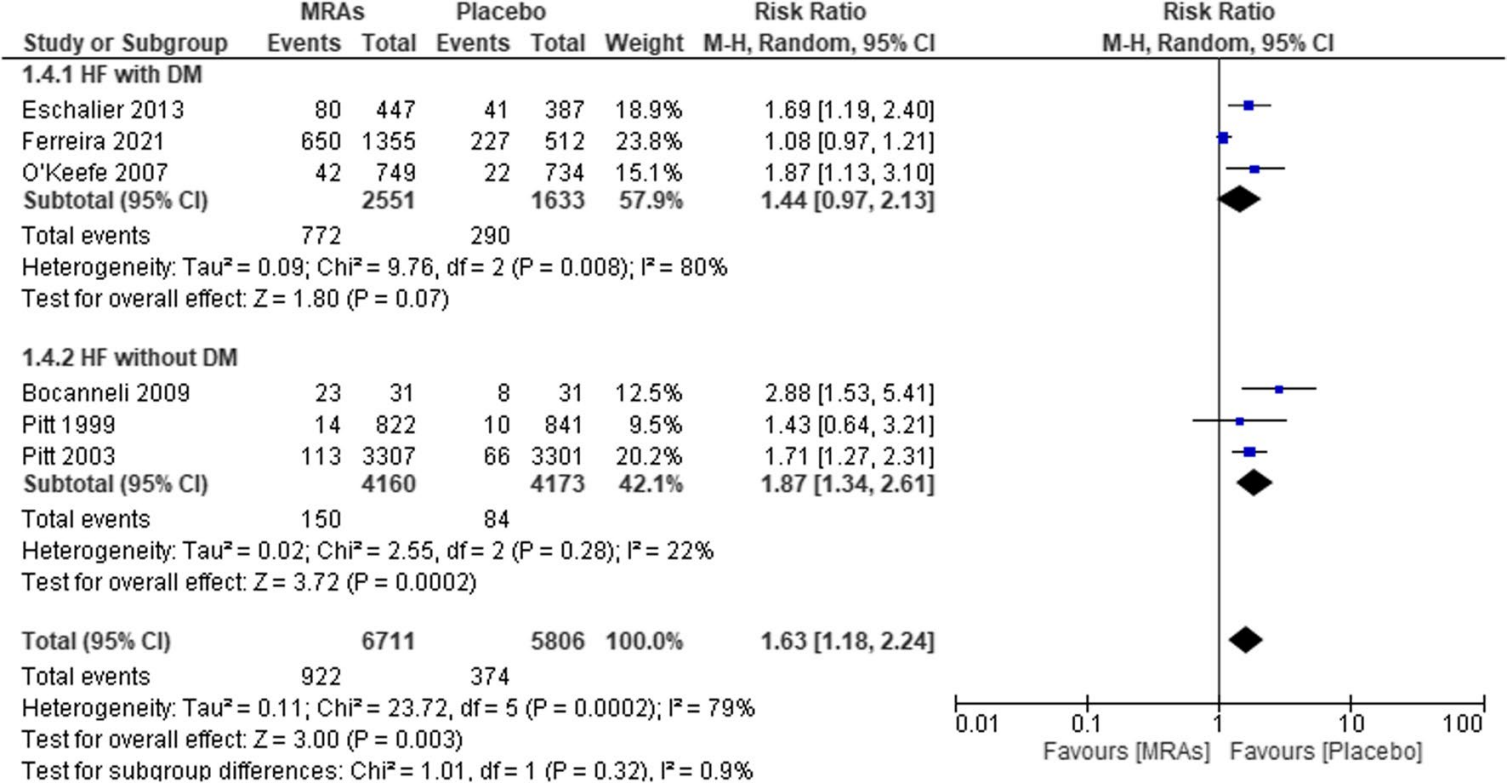
Forest plot of MRAs vs placebo for hyperkalemia

Hyperkalemia risk ratios in heart failure patients with and without diabetes mellitus, as well as those receiving placebo or MRAs, were evaluated in five studies (Fig. 7). We found that the combined analysis showed that the risk was “1.63 (95% CI 1.18–2.24, *P* = 0.003, *I*^2^ = 79%)” greater in the placebo group than in the MRA group. Patients with diabetes and heart failure who take a placebo are at an increased risk of hyperkalemia (RR 1.44, 95% CI 0.97–2.13; *P* = 0.07, *I*^2^ = 80%). If heart failure patients without diabetes were to receive a placebo, the risk of hyperkalemia would be much greater (RR 1.87, 95% CI 1.34–2.61; *P* = 0.0002, *I*^2^ = 22%).

## Discussion

In patients with DM, heart failure presents unique challenges due to the systemic effects of hyperglycemia and insulin resistance. Diabetic cardiomyopathy, a condition where heart muscle damage occurs independently of coronary artery disease or hypertension, plays a significant role in these challenges [41]. This condition is driven by several mechanisms. Chronic hyperglycemia leads to the formation of advanced glycation end-products (AGEs), which impair cardiac function by increasing myocardial stiffness and promoting fibrosis [42]. Additionally, diabetes is associated with chronic inflammation, exacerbating endothelial dysfunction and promoting atherosclerosis, both of which further impair heart function [43]. Autonomic dysfunction is another complication often seen in diabetic patients, where impaired regulation of the sympathetic and parasympathetic nervous systems leads to inadequate control of heart rate and vascular tone, contributing to heart failure progression [44]. Furthermore, microvascular complications, including capillary rarefaction and diabetic microvascular disease, reduce the heart’s ability to receive sufficient oxygen and nutrients, compounding the damage [45].

Steroidal MRAs, such as spironolactone and eplerenone, significantly decreased the risk of death from any cause, cardiovascular death, and cardiovascular mortality in patients hospitalized with HF in both the HF with DM and non-DM (N-DM) groups. A total of 15,272 participants were drawn from 10 randomized controlled trials. There was no difference in efficacy between 25 mg of spironolactone and 25 mg of MRA eplerenone. Research has shown that MRAs are effective, much more so than our meta-analysis. According to one study, individuals with chronic heart failure had reduced left ventricular systolic performance and cardiovascular mortality due to the use of eplerenone instead of spironolactone [46]. However, another RESEARCH study showed that both eplerenone and spironolactone reduced cardiovascular mortality and hospitalization in heart failure patients [47]; thus, spironolactone may have little advantage in certain measures. When comparing spironolactone with eplerenone in a cohort study from 2023, Larson et al. discovered no statistically significant differences in clinical outcomes; however, they did find disparities in medication adherence and dosage [48]. Furthermore, a network meta-analysis of RCTs and MRAs indicated that they successfully decreased cardiovascular and all-cause mortality in patients with heart failure [49].

The effects of spironolactone and eplerenone on all-cause mortality in HF patients with DM and N-DM have been extensively studied. Both MRAs significantly reduce all-cause mortality in HF patients, regardless of diabetes status. Naser et al. showed that eplerenone reduces cardiovascular mortality and improves left ventricular function more effectively than spironolactone in some cases [46]. Steroidal MRAs, such as spironolactone and canrenone, play crucial roles in the treatment of HF patients with and without DM. Spironolactone is metabolized in the liver to its active metabolite canrenone, which has a longer half-life and contributes to its prolonged therapeutic effects. Studies indicate that the effectiveness of spironolactone in reducing all-cause mortality in HF patients is significant, but its benefits may vary between diabetic and non-diabetic patients due to differences in metabolism and drug response [47, 50]. In HF patients with diabetes, the pharmacokinetics of MRAs can be affected by altered renal function, which may necessitate dose adjustments to avoid hyperkalemia and other adverse effects. Studies suggest that while both spironolactone and eplerenone are effective, their safety profile is crucial for determining the appropriate treatment for diabetic patients [51, 52]. Overall, the therapeutic effectiveness of spironolactone and canrenone in reducing all-cause mortality in HF patients with DM/N-DM is supported by their ability to reduce cardiovascular mortality and improve heart function, although careful monitoring and dose adjustments are essential to manage potential side effects in diabetic patients [49, 53].

Preventing CV death or mortality in HF patients with DM or N-DM using MRAs such as spironolactone and eplerenone has been the focus of several studies. These studies highlighted that both MRAs significantly reduce the risk of CV mortality and hospitalization due to HF. For instance, a RESEARCH trial confirmed that both spironolactone and eplerenone are effective at reducing all-cause and cardiovascular mortality in HF patients [47]. Another study revealed that, compared with spironolactone, eplerenone significantly improved left ventricular function and reduced cardiovascular mortality [46]. Moreover, a nationwide cohort study demonstrated that both spironolactone and eplerenone had comparable outcomes in reducing all-cause death and hospitalization in patients with HF, although eplerenone was associated with a slightly better adherence rate [48]. Additionally, studies have shown that MRAs effectively reduce mortality and hospitalization in HF patients with diabetic kidney disease when combined with ACE inhibitors or ARBs [54]. The effectiveness of eplerenone in preventing CV death and improving systolic function has also been documented in a randomized controlled trial [52]. Furthermore, comparative studies highlight that eplerenone might have a better safety profile concerning glucose homeostasis than spironolactone [23]. Overall, these findings underscore the importance of MRAs in managing HF and reducing CV mortality, with both spironolactone and eplerenone showing substantial benefits.

Hyperkalemia in HF patients with DM or N-DM due to the use of MRAs, specifically spironolactone and eplerenone, has been a significant concern in clinical practice. Studies have shown that these MRAs, while beneficial for reducing cardiovascular mortality and hospitalization, increase the risk of hyperkalemia, particularly in patients with renal dysfunction or those also receiving other renin–angiotensin system inhibitors. For instance, a meta-analysis revealed that hyperkalemia was more frequent in patients treated with MRAs than in patients treated with a placebo [55]. Another study highlighted that spironolactone significantly increased serum potassium levels in HF patients, with higher incidences of severe hyperkalemia than eplerenone [51]. Additionally, a systematic review revealed that both MRAs increase the risk of hyperkalemia, necessitating careful monitoring, especially in patients with chronic kidney disease (CKD) and DM [56]. Another meta-analysis confirmed that MRAs were associated with a higher risk of hyperkalemia but also demonstrated significant cardiovascular benefits [47]. Furthermore, studies such as those by Memon and Iqbal (2022) emphasized that newer non-steroidal MRAs such as finerenone might offer similar benefits with a decreased risk of hyperkalemia [57]. Additionally, long-term studies on spironolactone in patients with HF and CKD highlighted the need for stringent monitoring to manage the risks associated with hyperkalemia [58]. Finally, the importance of patient-specific risk factors such as baseline potassium levels and renal function in predicting hyperkalemia has been underscored in clinical practice [59]. While MRAs have demonstrated efficacy in reducing mortality and cardiovascular events in heart failure patients, their safety profile in diabetic patients with additional risk factors, such as renal impairment, remains a concern. This has led to the exploration of alternative therapies, including non-steroidal MRAs (nsMRAs) like finerenone, which offer a kidney-protective effect and a lower risk of hyperkalemia [60]. In particular, finerenone has been shown to reduce urinary albumin-to-creatinine ratio (uACR) and lower the incidence of adverse outcomes such as heart failure hospitalizations, stroke, and kidney failure [60, 61]. Therefore, in heart failure patients with diabetes and concurrent CKD, nsMRAs provide a promising alternative to traditional MRAs, offering both cardiovascular and renal protection while minimizing the risk of hyperkalemia. This highlights the need for further research and individualized treatment strategies in this population to address these gaps in care.

In general, the use of spironolactone and eplerenone in HF patients with and without DM has been extensively studied. Spironolactone, though effective in treating heart failure, is associated with endocrine-related side effects such as gynecomastia and menstrual irregularities, due to its interaction with androgen and progesterone receptors. Eplerenone, being more selective, causes fewer of these side effects, but both drugs carry a risk of hyperkalemia, which can become severe without proper monitoring [62, 63]. Hyperkalemia remains a major concern with both spironolactone and eplerenone, necessitating regular monitoring of serum potassium levels to avoid severe complications [64]. Future advancements may include optimizing dosing regimens and the development of more selective MRAs, such as finerenone, which could lower the risk of hyperkalemia while preserving efficacy, particularly in patients with heart failure and renal impairment. These studies also revealed that MRAs are effective at reducing all-cause mortality, CV death, and CV mortality from hospitalization for HF. However, these studies reported that using MRAs can increase the risk of hyperkalemia in both groups; similarly, Desai et al. reported that the use of MRAs also increases the risk of hyperkalemia, especially in patients with compromised renal function or those concurrently taking other renin–angiotensin system inhibitors [59]. Therefore, dose adjustments of MRAs are essential for HF patients with DM/N-DM, especially for HF patients with DM, to prevent side effects from treatment. These findings underscore the dual benefit and risk of MRA therapy in HF patients with and without diabetes, emphasizing the importance of personalized treatment and monitoring strategies to maximize therapeutic outcomes while minimizing adverse effects.

## Conclusions

The study findings indicate that MRAs significantly reduce the risk of all-cause mortality, death from cardiovascular causes, and cardiovascular mortality from heart failure hospitalization in both diabetic and non-diabetic heart failure patients. However, compared to placebo, MRAs significantly increased the risk of hyperkalemia in heart failure patients with and without diabetes. Additionally, the risk of hyperkalemia was notably higher in these patient subgroups when treated with MRAs. Overall, MRAs provide substantial benefits in mortality reduction but require careful monitoring for hyperkalemia risk in diabetic and non-diabetic heart failure patients.

## Data Availability

Data available within the article. The authors confirm that the data supporting the findings of this study are available within the article.

## Abbreviations

CI: Confidence interval
CV: Cardiovascular
CVD: Cardiovascular disease
DM: Diabetes mellitus
EF: Ejection fraction
EGFR: Estimated glomerular filtration rate
ESC: European Society of Cardiology
HbA1C: Hemoglobin A1C
HF: Heart failure
HFmrEF: Heart failure with mildly reduced ejection fraction
HFrEF: Heart failure with reduced ejection fraction
HOMA-IR: Homeostatic model assessment for insulin resistance
MRAs: Steroidal of mineralocorticoid receptor antagonists
N-DM: Non-diabetes mellitus
NO: Nitrite oxide
NYHA: New York Heart Association
PRISMA: Preferred Reporting Items for Systematic Review and Meta-Analysis
RAA: Renin–angiotensin–aldosterone
RCT: Randomized controlled trial
ROB: Risk of bias
RR: Risk ratio
T2DM: Type-2 diabetes mellitus
USA: United States of America
